# Measurement of lateral ventricle volume of normal infant based on magnetic resonance imaging

**DOI:** 10.1186/s41016-019-0156-9

**Published:** 2019-04-10

**Authors:** Zheng Lu, Jintao He, Yaxiong Yu, Zhicen Li, Zhi Li, Jian Gong

**Affiliations:** 1 Department of Pediatric Neurosurgery, Beijing Tiantan Hospital, Capital Medical University, Beijing, 100070 China; 2 Beijing Neurosurgical Institute, Capital Medical University, Beijing, 100070 China

**Keywords:** Infant, Lateral ventricle, Magnetic resonance imaging, Anthropometric measuring

## Abstract

**Background:**

Many neurophysiological diseases during infancy stage are associated with the morphology and size of the lateral ventricle. This research aims to measure the normal value range of lateral ventricle volume of normal infant and thus provide basic data for clinical treatment.

**Method:**

By retrospective analysis of magnetic resonance inspection (MRI) cranial image of 165 infants in the Department of Pediatric Neurosurgery, Beijing Tiantan Hospital, the infants were divided into four groups according to their age, including the first group (1~3 month, *n* = 12), the second group (4~6 month, *n* = 33), the third group (7~9 month, *n* = 51), and the fourth group (10~12 month, *n* = 69). On Neurosoft image workstation, it can measure the sectional area of the lateral ventricle volume at each layer of axis T2W image and calculate the lateral ventricle volume using the Cavalieri method. Moreover, the correlations between lateral ventricle volume and gender, side difference, and month age were analyzed.

**Results:**

95% confidence interval of total bilateral ventricle volume of normal infant: 11920.22~14,266.28 mm^3^ for male infant and 9922.22~12,263.17 mm^3^ for female infant; 95% confidence interval of left side ventricle volume: 6254.72~7546.94 mm^3^ for male infant and 5206.03~6479.99 mm^3^ for female infant; 95% confidence interval of right side ventricle volume: 5041.56~6743.29 mm^3^ for male infant and 4695.00~5804.40 mm^3^ for female infant. The lateral ventricle volume of the male infant was normally larger than that of the female infant (*p* < 0.05). For both male and female infants, the left side ventricle volume was larger than the right ventricle volume (*p* < 0.01). There was no significant difference in lateral ventricle volume between infants over 3 months old.

**Conclusion:**

The normal value range of lateral ventricle volume of the infant can be obtained via referring MRI image. The lateral ventricle volume of infant varies upon gender and ventricle side.

## Background

Infant refers to a child with age from just born to 1-year-old. During infant period, many neuropsychiatric disorders are associated with the morphology and size of lateral ventricle [1, 2], wherein lateral ventricle volume is a key parameter, which has been reported in Australia, UK, and the USA, basing on few cases [1, 3, 4]. The purpose of this research is to measure the normal value range of lateral ventricle volume of a normal infant and thus to provide the basis for clinical research.

## Methods

### General data

From 2010 to 2018, there were totally 2091 infants accepting MRI cerebral examination in our hospital, in which 165 infants had normal image results. A retrospective analysis of these data was performed in this work. The 165 infants were collected from the Department of Neurosurgery or Department of Pediatrics, including 102 male infants and 63 female infants, with age varying within 2–12 months. They visited the hospital for mild head injury, physical examination, or scalp tumor.

### Image collection

Four magnetic resonance machines of our hospital were used for random data collection, with a field intensity of 1.5–3.0 T, layer thickness of 4–5 mm, and interlamellar space of 0.5–1.5 mm. The scanning sequence at least included normal SE T1W and T2W, and some included fluid-attenuated inversion recovery (FLAIR) or DWI, which were useful for intracranial lesion identification. The scanning results were read and judged by two neuroimaging specialists. The image data were analyzed and calculated on Neurosoft workstation. The inclusion criteria are as follows: (1) no intracranial abnormalities, (2) smooth process of labor and normal development, (3) no family genetic diseases. The exclusion criteria are as follows: (1) skull fractures; (2) there are some mutations that may affect lateral ventricle volume, such as large cisterna magna, the fifth ventricle, and arachnoid cysts; (3) the image is involved with artifact or fuzziness; and (4) suspicious history of epilepsy.

### Measuring and calculating method of lateral ventricle volume

**Fig. 1:**
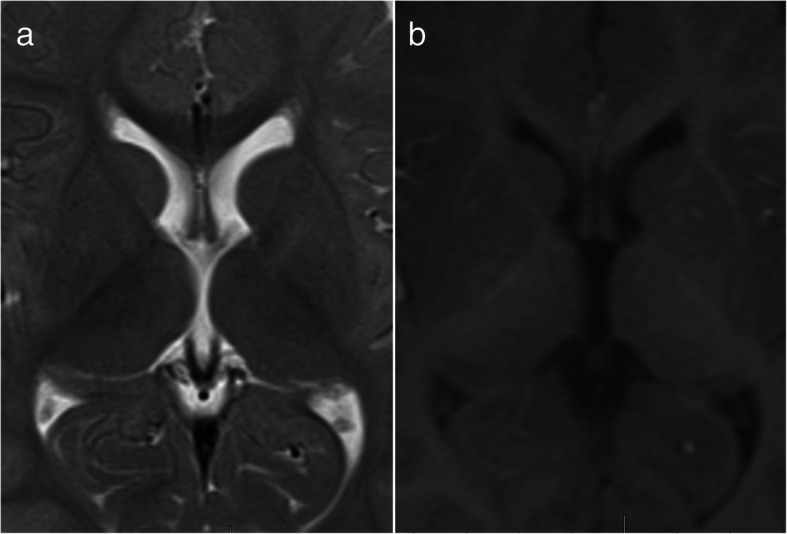
Morphology of lateral ventricle on T2W (**a**) and T1W (**b**) image. The internal cerebral vein on T2W image is clear

**Fig. 2:**
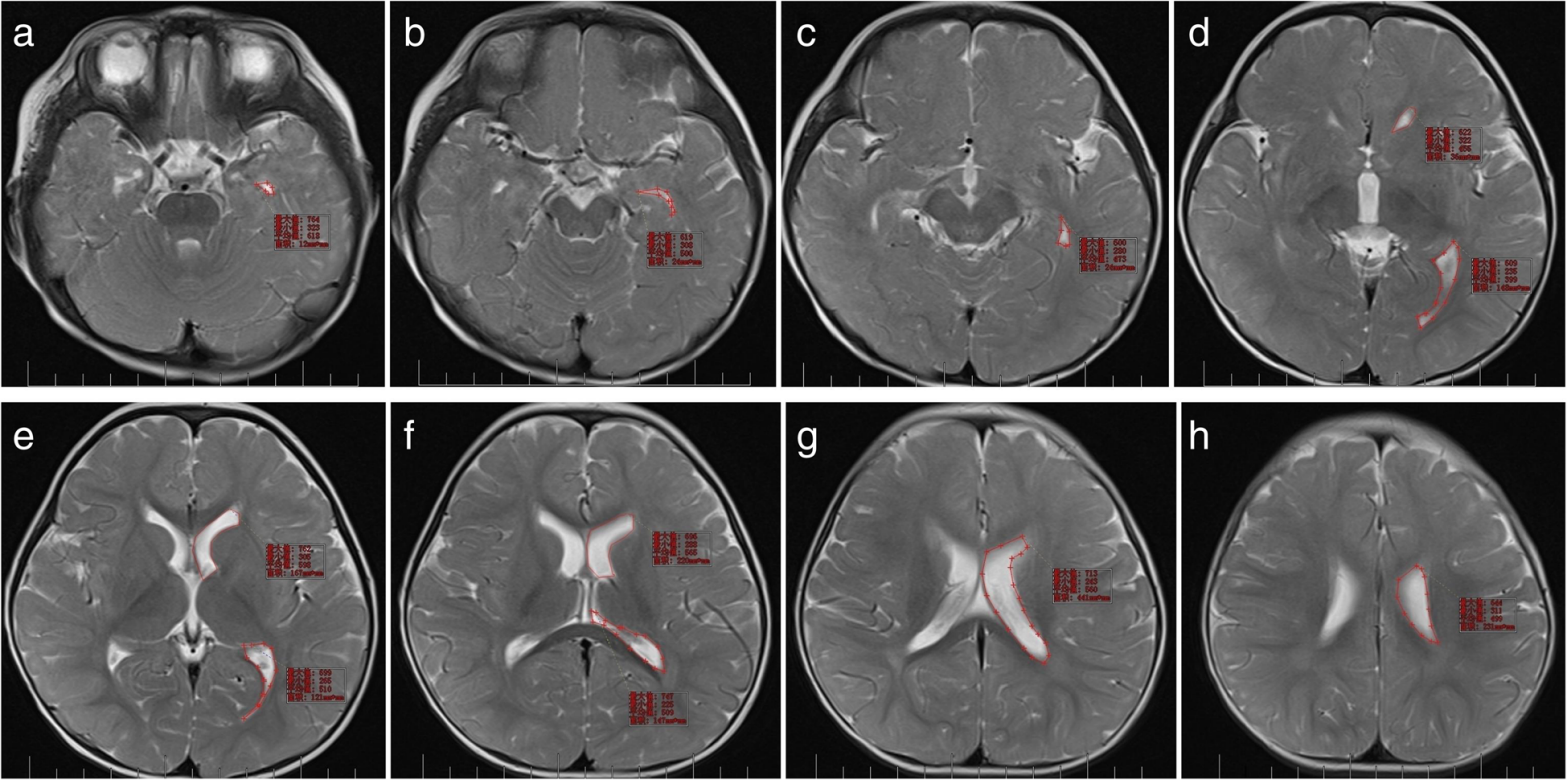
**a**–**h** Sketching ROI on lateral ventricle containing layer. The area of ROI is automatically revealed by workstation (**a** pons, **b** suprasellar cistern, **c** midbrain, **d** diaterma, **e** upper portion of the third ventricle, **f** diatela, **g** body of the lateral ventricle and posterior horn of the lateral ventricle, **h** upper portion of the lateral ventricles)

First, adjust the window width and window position to make the comparison of image as clear as possible. Then, select T2W axial image, and manually sketch the boundaries of lateral ventricle on each lateral ventricle containing layer using built-in tools of the working station, which is the so-called region of interest (ROI). After finishing sketching, the workstation will automatically show the area of such area. Finally, calculate the lateral ventricle volume using the Cavalieri method by multiplying the sum of sectional areas of lateral ventricles with the layer thickness (Figs 1 and 2).

### Statistical analysis

Using SPSS19.0 statistical software, the lateral ventricle volumes of infants with different gender, ventricle sides, and age were subjected to normality test and homogeneity test of variance. The 95% confidence interval was selected from the normal value range of lateral ventricle volume. The comparison of total lateral ventricle volume between different genders was subjected to independent sample *t* test. The comparison of total lateral ventricle volume between different ventricle sides was subjected to pairing *t* test. The comparison of total lateral ventricle volume between different ages was subjected to one-way analysis of variance. The difference was of statistical significance when *p* < 0.05.

## Results

### Measurement of volume

The total bilateral ventricle volume of 165 normal infants was 12,329.41 ± 5575.04 mm^3^. The lateral ventricle volumes of infants with different gender and ventricle sides as well as 95% confidence interval are shown in Table 1. The total lateral ventricle volumes of infants with different age are shown in Table 2.

**Table 1:** Lateral ventricle volume of 165 normal infants and 95% confidence interval (mm^3^)

Gender	Bilateral		Left side		Right side	
	Volume	95% confidence interval	Volume	95% confidence interval	Volume	95% confidence interval
Male	13,093.25 ± 5972.09	11,920.22~14,266.28	6900.83 ± 3289.44	6254.72~7546.94	6192.43 ± 2804.55	5041.56~6743.29
Female	11,092.70 ± 4647.55	9922.22~12,263.17	5843.01 ± 2529.24	5206.03~6479.99	5249.69 ± 2202.58	4695.00~5804.40
Lenene homogeneity test of variance	*F* = 2.854, *p* = 0.093		–	–	–	–
Independent sample *t* test	*t* = 2.267, *p* < 0.05		–	–	–	–
Paired sample *t* test	–		Male: *t* = 5.748, *p* < 0.01			
			Female: *t* = 4.972, *p* < 0.01			

After implementation of Lenene homogeneity test of variance for both groups of data (*F* = 2.854, *p* = 0.093), the independent sample *t* test was conducted for both groups. The total lateral ventricle volume of the male infant was larger than that of the female infant, wherein the difference was of statistical significance (*t* = 2.267, *p* < 0.05). For both male and female infants, the left side ventricle volume was larger than the right ventricle volume based on paired sample *t* test, wherein the difference was of statistical significance (male: *t* = 5.748, *p* < 0.01; female: *t* = 4.972, *p* < 0.01)

**Table 2:** Lateral ventricle volume of 165 normal infants with different age (mm^3^)

Group (month age)	Mean month age	Gender	Number of infants	Total lateral ventricle volumes (mm^3^)
1~3 (*n* = 12)	2.5	Male	7	10,359.00
		Female	5	8994.60
4~6 (*n* = 33)	5.2 ± 0.8	Male	22	12,174.41 ± 6160.34
		Female	11	9273.54 ± 3462.47
7~9 (*n* = 51)	8.1 ± 0.8	Male	32	13,308.13 ± 4815.42
		Female	19	12,625.47 ± 5382.09
10~12 (*n* = 69)	11.5 ± 0.8	Male	41	13,885.41 ± 7027.05
		Female	28	11,141.93 ± 4328.60
One-way analysis of variance	Male: *F* = 0.915, *p* > 0.05; female *F* = 1.641, *p* > 0.05			
	Male: *F* = 0.552, *p* > 0.05; female *F* = 1.899, *p* > 0.05			

Using one-way analysis of variance, the difference of lateral ventricle volume between male and female infant was of no statistical significance (male: *F* = 0.915, *p* > 0.05; female: *F* = 1.641, *p* > 0.05), considering that the number of infants with age lower than 3 months was only 12, which was too less. Removing such group, the differences among the other three groups were still of no statistical significance (male: *F* = .552, *p* > 0.05; female: *F* = 1.899, *p* > 0.05)

### Comparison of total lateral ventricle volume between different gender

After implementing Levene homogeneity test of variance for data of both groups (*F* = 2.854, *p* = 0.093), independent sample *t* test was conducted. The lateral ventricle volume of the male infant was larger than that of the female infant, wherein the difference was of statistical significance (*t* = 2.267, *p* < 0.05).

### Comparison of lateral ventricle volume between different ventricle sides

Based on paired sample *t* test, the left side ventricle volume was larger than that of the right side ventricle volume for both genders, wherein the difference was of statistical significance (male: *t* = 5.748, *p* < 0.01; female: *t* = 4.972, *p* < 0.01).

### Comparison of lateral ventricle volumes between different gender and different month age

Based on one-way analysis of variance, the difference of lateral ventricle volume between male and female infant was of no statistical significance (male: *F* = 0.915, *p* > 0.05; female: *F* = 1.641, *p* > 0.05), considering the number of infants with age under 3 months old was only 12, which was too less. Removing such group, the difference among the other three groups was still of no statistical significance (male: *F* = 0.552, *p* > 0.05; female *F* = 1.899, *p* > 0.05).

## Discussion

In diagnosis and treatment of pediatric neurosurgery, it often involves lateral ventricle systems, such as congenital hydrocephalus and obstructive hydrocephalus. Lateral external drainage and ventricular irrigation are common treatment approaches [5–7]. Literatures on lateral ventricle volume of normal children especially infants are very few [8].

Lateral ventricle consists of frontal angle, temporal angle, occipital angle, and trigonum, which is an irregular body. Many human tissues or organs are in irregular morphology. For a long period of time, it is very difficult to calculate the volumes of these organs. The Cavalieri method is a method to obtain the quantitative information of 3D spatial structure based on 2D image data. To be specific, segment the irregular body into several parallel layers according to the set thickness (*h*), and the sectional area of each layer is expressed by *S*. The irregular body volume (*V*) can be calculated by multiplying the sum of sectional areas of all layers (∑*S*) with the thickness (*h*), i.e., *V* = ∑*S* × *h*. Only requiring that all layers are parallel and equidistant with each other, the results calculated by the Cavalieri method are free from the influence of cutting angle. MRI is a parallel and equidistant scanning image, which just meets the requirements of the Cavalieri method. Moreover, MRI enjoys a high distinguishing capacity for brain tissue, which is a very ideal research method [9]. In 1996, Garden et al. first measured the cranial capacity of the infant using MRI according to the Cavalieri method [10]. Gilmore et al. believed 3D ultrasound is a fast and reliable method which can be used to measure infant lateral ventricle volume via anterior fontanel but also recognized that MRI remained to be a “gold standard” [1]. Since the morphology of lateral ventricle was displayed through cerebrospinal fluid (CSF) signal, T2W image is sensitive to water molecule signal and can clearly show the vein of septum pellucidum, the cerebral internal vein, which can accurately sketch out ROI (Fig. 1).

The results of this research showed that the normal value range of total lateral ventricle volume of the male infant was 11,920.22~14,266.28 mm^3^, which was larger than that (9922.22~12,263.17 mm^3^) of the female infant (*p* < 0.05). The lateral ventricle of adult people also follows such rule [11]. Holland et al. found that the brain capacity of a newborn was roughly 1/3 of an adult’s brain capacity, without difference upon gender. Since birth, the increasing rate of brain capacity of the male infant (200.4 mm^3^ per day) was faster than that of the female infant. When growing till the third month, the brain capacity of the male infant was significantly larger than that of the female infant. The brain capacity of the male adult is also larger than that of the female adult [12]. The difference in lateral ventricle volume of the infants between genders is probably associated with the difference in brain capacity between genders.

This research showed that the left side ventricle volume was larger than the right side ventricle volume for both male and female infants (*p* < 0.01). Former studies found that the left side ventricle volume was larger than the right side ventricle volume when the infant was just born [13]. For the 2-month-old infants, the left side ventricle volume is averagely larger than the right side ventricle volume by 462 mm^3^ [6]. The left side ventricle volume of the normal adult is also larger than its right side ventricle volume [10]. This research further proves such a result that the asymmetry between the left and right ventricle volume is inherent and lifelong. Not only ventricle, but thalamus and hippocampus also have such asymmetry [14]. This is a key property of the human brain, which may be associated with the functional specialization of the left or right brain hemispheres.

This research showed that 3 months after the infant was born, there was no significant difference in lateral ventricle volume regardless of gender. For infants who are 3 months old, there was no significant increase of lateral ventricle volume. Holland et al. found that within the 3 months after birth, the increasing rate of cranial capacity was about 1% per day; when growing to the end of the third month, the cranial capacity has been increased by 64%, which was more than half of an adult’s cranial capacity. After that, the increasing rate decreased to about 0.4% per day [8]. Bompard et al. believed that bilateral ventricular volume increased most significantly within 6 months after birth, which was synchronized with the increase of brain volume. Although brain volume was still increasing from 1 to 2 years old, the increase of lateral ventricular volume slowed down significantly [15].

There is a correlation between lateral ventricle volume and cranial capacity. The results of this research are consistent with the features of infant brain development.

The significance of this study is to provide a new observational index for the prediction and early diagnosis of certain diseases, especially congenital hydrocephalus. In the past, linear indexes were more often used to determine the size of the ventricle, such as Evans’ index, frontal horn index, and bicaudate index, while in recent years, the frontal-occipital horn ratio (FOHR) has been adopted [16]. These indexes can reflect the ratio of lateral ventricular volume to brain volume but cannot directly reflect the size of the lateral ventricle. Brain tissue develops rapidly in infancy, while lateral ventricular volume and brain volume do not develop simultaneously. There are definitely limitations in the diagnosis of hydrocephalus by using linear indexes. It may be a new diagnostic approach to measure the volume of a baby’s lateral ventricle and then compare it with the normal value range. It should be pointed out that the volume of the lateral ventricle of an infant is only an index; still, a comprehensive judgment should be made based on the medical history, physical signs, and imaging characteristics.

Study on infants is different from that on adults. Recruitment of healthy volunteers can be performed for the study on adult people, while it is impossible for the study on infant. The unique point of this research is that all examinations are based on retrospective data, with different images collecting MRI devices of different settings. The process of sketching ROI is also affected by some subjective factors. However, the results of this research are consistent with those of former researches, which can reflect a certain feature of infant lateral ventricle and provide certain reference basis for clinical diagnosis and treatment. With the accumulation of data as well as the improvement of image processing technology, more accurate data will be obtained.

## Conclusion

The normal value range of the lateral ventricle volume of an infant can be obtained via referring MRI images. The lateral ventricle volume of infant varies upon gender and ventricle side. These data can serve as a basis for prognosis of hydrocephalus.

## Abbreviations

3D: Three-dimensional
CSF: Cerebrospinal fluid
DWI: Diffusion-weighted imaging
FLAIR: Fluid-attenuated inversion recovery
FOHR: Frontal occipital horn ratio
MRI: Magnetic resonance imaging
ROI: Region of interest
T1W: T1-weighted
T2W: T2-weighted
